# Battery of behavioral tests in mice to study postoperative delirium

**DOI:** 10.1038/srep29874

**Published:** 2016-07-20

**Authors:** Mian Peng, Ce Zhang, Yuanlin Dong, Yiying Zhang, Harumasa Nakazawa, Masao Kaneki, Hui Zheng, Yuan Shen, Edward R. Marcantonio, Zhongcong Xie

**Affiliations:** 1 Department of Anesthesia, Zhongnan Hospital of Wuhan University, Wuhan, 430071, P. R. China; 2 Geriatric Anesthesia Research Unit, Department of Anesthesia, Critical Care and Pain Medicine, Massachusetts General Hospital and Harvard Medical School, Charlestown, MA 02129-2060, USA; 3 Department of Anesthesia, China-Japan Union Hospital of Jilin University, Changchun, Jilin, 130033, P. R. China; 4 Department of Anesthesia, Critical Care and Pain Medicine, Massachusetts General Hospital, Shriners Hospitals for Children and Harvard Medical School, Charlestown, MA 02129-2060, USA; 5 Massachusetts General Hospital Biostatistics Center, Massachusetts General Hospital and Harvard Medical School, Boston, MA 02114, USA; 6 Department of Psychiatry, Tenth People’s Hospital of Tongji University, Shanghai, 200072, P. R. China; 7 Divisions of General Medicine and Primary Care and Gerontology, Department of Medicine, Beth Israel Deaconess Medical Center and Harvard Medical School, Boston, MA 02215.

## Abstract

Postoperative delirium is associated with increased morbidity, mortality and cost. However, its neuropathogenesis remains largely unknown, partially owing to lack of animal model(s). We therefore set out to employ a battery of behavior tests, including natural and learned behavior, in mice to determine the effects of laparotomy under isoflurane anesthesia (Anesthesia/Surgery) on these behaviors. The mice were tested at 24 hours before and at 6, 9 and 24 hours after the Anesthesia/Surgery. Composite Z scores were calculated. Cyclosporine A, an inhibitor of mitochondria permeability transient pore, was used to determine potential mitochondria-associated mechanisms of these behavioral changes. Anesthesia/Surgery selectively impaired behaviors, including latency to eat food in buried food test, freezing time and time spent in the center in open field test, and entries and duration in the novel arm of Y maze test, with acute onset and various timecourse. The composite Z scores quantitatively demonstrated the Anesthesia/Surgery-induced behavior impairment in mice. Cyclosporine A selectively ameliorated the Anesthesia/Surgery-induced reduction in ATP levels, the increases in latency to eat food, and the decreases in entries in the novel arm. These findings suggest that we could use a battery of behavior tests to establish a mouse model to study postoperative delirium.

Postoperative delirium, an acute, transient, fluctuating disturbance in attention, cognition, and level of consciousness, is a common (15–53%) postoperative complication^1^,^2^,^3^, and it can lead to 2- to 20-fold increase in mortality^4^, long term functional impairment, postoperative cognitive dysfunction, and increased costs of medical care [5,6,7,8, reviewed in^9^,^10^]. However, at the present time, postoperative delirium remains a wholly clinical diagnosis; its causes, neuropathogenesis and targeted intervention(s) remain largely to be determined. One of the barriers to advancing work on the basic mechanisms of postoperative delirium is the lack of animal model(s).

Thus far, there are only a few animal models for delirium research. Specifically, the “paddling” T-maze alternation task has been used to study the effects of bacterial endotoxin lipopolysaccharide on behavior changes in rodents^11^. Culley *et al*. have reported the attention set-shifting task as a potential animal model to study delirium^12^. Both of these studies tested the learned behaviors of rodents, suggesting the abnormalities observed may be indicative of higher order executive dysfunction and may not be directly relevant to the more basic dysfunction seen in delirium.

The Confusion Assessment Method (CAM) algorithm is widely utilized to determine the presence of delirium in patients^13^. CAM consists of four clinical features: (1) acute onset and fluctuating course, (2) inattention, (3) disorganized thinking, and (4) altered level of consciousness. These features occur naturally without learning. CAM includes many tests to determine the changes in behavior. Thus, a single behavior test in animals may not completely cover the aspects of delirium and may not be consistent with the concept of CAM. We therefore set out to establish a battery of behavioral tests (buried food test, open field test and Y maze test) to assess changes in both natural and learned behaviors following the surgery (simple laparotomy) under isoflurane anesthesia (Anesthesia/Surgery) in mice. We employed buried food test and open field test to assess the changes in natural behavior and we used Y maze test to assess the changes in learned behavior of mice. Note that these tests could assess the behaviors in mice that are dependent on the presence and intactness of attention, thought organization, and a normal level of consciousness. Therefore, these tests may evaluate certain aspects of delirium analogous to the CAM features. We performed these tests preoperatively (24 hours before the Anesthesia/Surgery), and again at 6, 9 and 24 hours after the Anesthesia/Surgery. Serial testing enabled us to assess the potential acute onset and fluctuating course, which is the first CAM feature. Finally, we calculated a composite Z score for each mouse to represent changes of the sum [normalized with the standard deviation (SD) for that sum in the controls] of these behaviors.

As proof of concept that our mouse model would be useful in mechanistic studies of delirium, we investigated whether the Anesthesia/Surgery could change the brain level of ATP and reactive oxygen species (ROS), and whether cyclosporine A (CsA), an inhibitor of mitochondria permeability transient pore (mPTP), could rescue the Anesthesia/Surgery-induced biochemical and behavioral changes. The primary hypothesis in the current study is that Anesthesia/Surgery in mice induces changes in natural and learned behaviors associated with delirium.

## Results

### Time-dependent effects of Anesthesia/Surgery on the behavior of mice in buried food test

**Figure 1:**
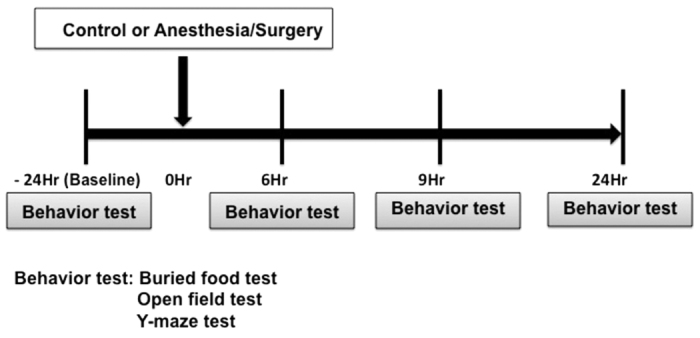
Diagram of the experimental design. The mice received behavior tests at 24 hours (baseline) before the abdominal surgery under isoflurane anesthesia (Anesthesia/Surgery), and then at 6, 9 and 24 hours after the Anesthesia/Surgery.

We set out to assess the effects of abdominal surgery under isoflurane anesthesia (Anesthesia/Surgery) on mouse behavior changes in buried food test, open field test and Y maze test at 24 hours before the Anesthesia/Surgery, and then 6, 9 or 24 hours after the Anesthesia/Surgery (Fig. 1).

**Figure 2:**
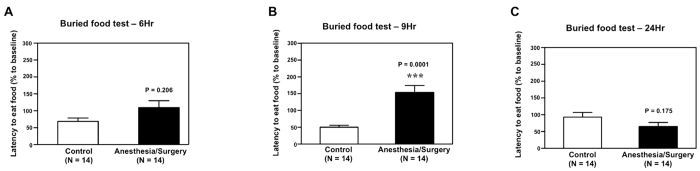
Anesthesia/Surgery impairs the behavior of mice in buried food test in a time-dependent manner. (**A**) Anesthesia/Surgery (black bar) does not significantly change the latency to eat food of the mice in the buried food test as compared to the control condition (white bar) at 6 hours after the Anesthesia/Surgery. (**B**) Anesthesia/Surgery (black bar) significantly increases the latency to eat food of the mice in the buried food test as compared to the control condition (white bar) at 9 hours after the Anesthesia/Surgery. (**C**) Anesthesia/Surgery (black bar) does not significantly change the latency to eat food of the mice in the buried food test as compared to the control condition (white bar) at 24 hours after the Anesthesia/Surgery. N = 14 in the control condition group and N = 14 in the Anesthesia/Surgery group.

We first assessed whether the Anesthesia/Surgery could impair the natural behaviors of mice by employing buried food test and open field test. As can be seen in Fig. 2, the Anesthesia/Surgery (black bar) increased the latency of mice to eat the food as compared to the control condition (white bar) in buried food test at 9 hours after the Anesthesia/Surgery: 153% versus 50%, P = 0.0001 (Fig. 2B). The Anesthesia/Surgery did not significantly alter the latency of mice to eat food as compared to the control condition at 6 (Fig. 2A) or 24 (Fig. 2C) hours after the Anesthesia/Surgery. These data suggest that the Anesthesia/Surgery was able to impair the mice’s abilities to find and eat the food, and this impairment was time-dependent.

### Time-dependent effects of Anesthesia/Surgery on the behavior of mice in open field test

Next, we assessed the effects of the Anesthesia/Surgery on open field behavior in the mice. The Anesthesia/Surgery did not significantly change the total distance traveled by mice in the open field test as compared to the control condition at 6 (Supplemental information Fig. 1A1), 9 (Supplemental information Fig. 1A2) or 24 (Supplemental information Fig. 1A3)] hours after the Anesthesia/Surgery. These data suggest that the Anesthesia/Surgery did not impair the motor function of the mice.

**Figure 3:**
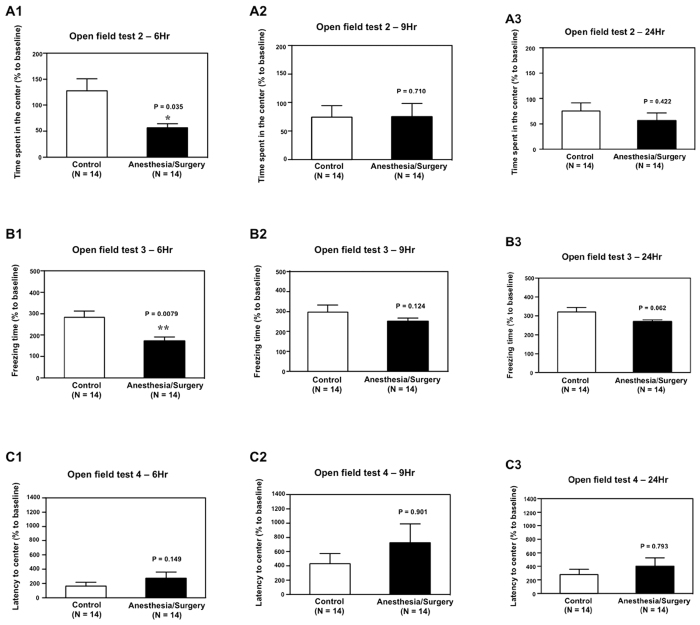
Anesthesia/Surgery impairs the behavior of mice in open field test in a time-dependent manner. (**A)** Anesthesia/Surgery (black bar) significantly decreases the time spent in the center of the open field as compared to the control condition (white bar) at 6 (**A1**), but neither 9 (**A2**) nor 24 (**A3**), hours after the Anesthesia/Surgery in the mice. (**B**) Anesthesia/Surgery (black bar) significantly decreases the freezing time in the open field test as compared to the control condition (white bar) at 6 (**B1**), but neither 9 (**B2**) nor 24 (**B3**), hours after the Anesthesia/Surgery in the mice. (**C)** Anesthesia/Surgery (black bar) does not significantly change the time to reach the center (latency to the center) in the open field test as compared to the control condition (white bar) at 6 (**C1**), 9 (**C2**) and 24 (**C3**) hours after the Anesthesia/Surgery in the mice. N = 14 in the control condition group and N = 14 in the Anesthesia/Surgery group.

Then, we found that the Anesthesia/Surgery (black bar) decreased the time spent in the center of the open field as compared to the control condition (white bar) in the mice at 6 hours after the Anesthesia/Surgery: 56% versus 128%, P = 0.035 (Fig. 3A1). The Anesthesia/Surgery did not significantly alter the time spent in the center of the open field as compared to the control condition in the mice at 9 (Fig. 3A2) or 24 (Fig. 3A3) hours after the Anesthesia/Surgery. The Anesthesia/Surgery (black bar) decreased the freezing time of mice in the open field test as compared to the control condition (white bar) at 6 hours after the Anesthesia/Surgery: 122% versus 282%, P = 0.0079 (Fig. 3B1). The Anesthesia/Surgery did not significantly alter the freezing time of mice in the open field test as compared to the control condition in the mice at 9 (Fig. 3B2) or 24 (Fig. 3B3) hours after the Anesthesia/Surgery. Finally, the Anesthesia/Surgery did not significantly alter the mice’s time to the center (latency) in the open field test as compared to the control condition at 6 (Fig. 3C1), 9 (Fig. 3C2) or 24 (Fig. 3C3) hours after the Anesthesia/Surgery. Collectively, these data suggest that the Anesthesia/Surgery could disturb some open field behaviors (e.g., time spent in the center and freezing time), but not others (e.g., total distance and latency to the center), in mice, and moreover, such disturbances were time-dependent.

### Time-dependent effects of Anesthesia/Surgery on the behavior of mice in Y maze test

Given the findings that the Anesthesia/Surgery might impair the natural behaviors of the mice, we next asked whether the Anesthesia/Surgery could also impair learned behavior in mice by employing Y maze test. The Anesthesia/Surgery did not significantly alter the number of arm visits of the mice as compared to the control condition in the Y maze test at 6 (Supplemental information Fig. 2A1), 9 (Supplemental information Fig. 2A2) or 24 (Supplemental information Fig. 2A3) hours after the Anesthesia/Surgery.

**Figure 4:**
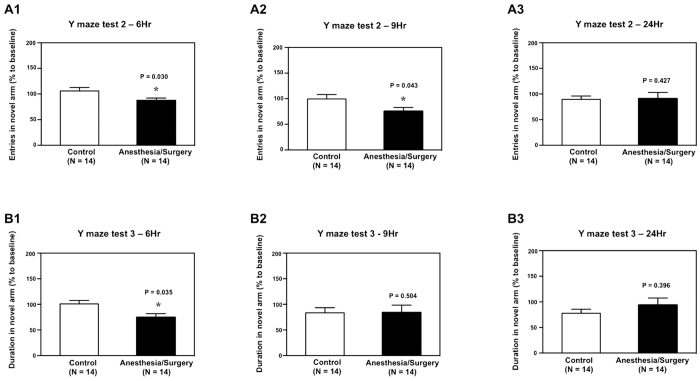
Anesthesia/Surgery impairs the behavior of mice in Y maze test in a time-dependent manner. (**A**) Anesthesia/Surgery (black bar) significantly decreases the number of entries in the novel arm in the Y maze test as compared to the control condition (white bar) at 6 (**A1**) and 9 (**A2**), but not 24 (**A3**), hours after the Anesthesia/Surgery in the mice. (**B**) Anesthesia/Surgery (black bar) significantly decreases the duration in the novel arm in the Y-maze test as compared to the control condition (white bar) at 6 (**B1**), but not 9 (**B2**) nor 24 (**B3**), hours after the Anesthesia/Surgery in the mice. N = 14 in the control condition group and N = 14 in the Anesthesia/Surgery group.

The Anesthesia/Surgery (black bar) reduced the number of mice’s entries in the novel arm as compared to the control condition (white bar) in the Y maze test at 6 hours: 87% versus 106%, P = 0.030 (Fig. 4A1), and 9 hours: 76% versus 100%, P = 0.043 (Fig. 4A2) after the Anesthesia/Surgery. The Anesthesia/Surgery did not significantly alter the number of mice’s entries in the novel arm as compared to the control condition in the Y maze test at 24 hours after the Anesthesia/Surgery (Fig. 4A3). Finally, the Anesthesia/Surgery (black bar) decreased the duration in the novel arm as compared to the control condition (white bar) in the Y maze test at 6 hours after the Anesthesia/Surgery: 75% versus 101%, P = 0.035 (Fig. 4B1), but not at 9 (Fig. 4B2) or 24 (Fig. 4B3) hours after the Anesthesia/Surgery in the mice. Taken together, these data suggest that the Anesthesia/Surgery could disturb some Y maze behaviors (e.g., entries in the novel arm and duration in the novel arm), but not others (e.g., number of arm visits), in mice in a time-dependent manner.

Collectively, the Anesthesia/Surgery impaired the natural (buried food test and open field test) and learned (Y maze test) behaviors of mice in a time-dependent manner (Table 1). Notably, the Anesthesia/Surgery only selectively impaired certain components of the behaviors, e.g., time spent in the center versus latency to the center in the open field test, and entries in the novel arm versus number of arm visits in the Y maze test (Table 1).

Composite Z scores for each of the 14 mice in the control and the Anesthesia/Surgery groups were calculated (Table 2). The composite Z score was more variable in the mice in control group, e.g., 6 and 8 of 14 composite Z scores were negative at 6 and 9 hours after the control treatment, respectively. However, the composite Z score was less variable in the mice of the Anesthesia/Surgery group, e.g., 2 and 0 of 14 composite Z scores were negative at 6 and 9 hours after the Anesthesia/Surgery, respectively. The composite Z scores were variable in the mice of both the control and Anesthesia/Surgery groups at 24 hours after the Anesthesia/Surgery (Table 2). Moreover, the mean of the composite Z score for mice in the Anesthesia/Surgery group was significantly greater (indicating worse performance) than that for mice in the control group at 6 (0.968 versus 0.000, P = 0.017) or 9 (1.539 versus 0.000, P = 0.0007), but not 24 (−0.054 versus 0.000, P = 0.906), hours after the Anesthesia/Surgery (Table 2). Taken together, these results further suggest that the Anesthesia/Surgery could induce significant behavioral impairment as compared to the control condition in a time-dependent manner, e.g., at 6 and 9, but not 24, hours after the Anesthesia/Surgery.

### CsA selectively ameliorated the Anesthesia/Surgery-induced ATP reduction and behavioral changes in mice

**Figure 5:**
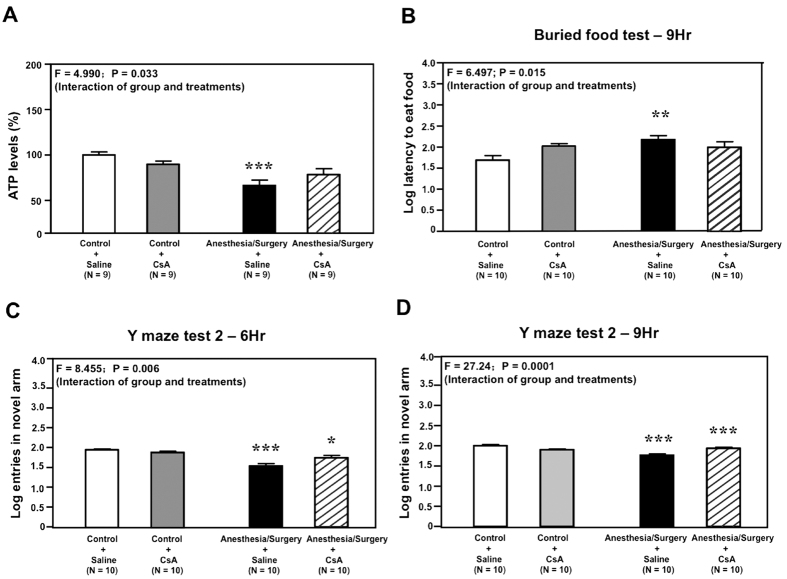
CsA mitigates the Anesthesia/Surgery-induced ATP reduction and behavior changes in buried food test and Y maze test. (**A**) Anesthesia/Surgery (black bar) decreases the ATP level in mouse brain tissue as compared to the control condition (white bar) immediately after the Anesthesia/Surgery. Treatment with CsA alone (gray bar) does not significantly change the ATP level as compared to the control condition (white bar). However, there is a significant interaction of CsA and Anesthesia/Surgery on the ATP level, and treatment with CsA attenuates the Anesthesia/Surgery-induced reduction in ATP level. (**B**) Anesthesia/Surgery (black bar) increases the latency to eat food of the mice in the buried food test as compared to the control condition (white bar) at 9 hours after the Anesthesia/Surgery. Treatment with CsA alone (gray bar) does not significantly change the latency as compared to the control condition (white bar). However, there is a significant interaction of CsA and Anesthesia/Surgery on the latency, and treatment with CsA attenuates the Anesthesia/Surgery-induced increase in the latency. (**C**) Anesthesia/Surgery (black bar) decreases the number of entries in the novel arm of Y maze test as compared to the control condition (white bar) at 6 hours after the Anesthesia/Surgery. Treatment with CsA alone (gray bar) does not significantly change the number of entries as compared to the control condition (white bar). However, there is a significant interaction of CsA and Anesthesia/Surgery on the number of entries, and treatment with CsA attenuates the Anesthesia/Surgery-induced decrease in the number of entries. (**D**) Anesthesia/Surgery (black bar) decreases the number of entries in the novel arm of Y-maze test as compared to the control condition (white bar) at 9 hours after the Anesthesia/Surgery. Treatment with CsA alone (gray bar) does not significantly change the number of entries as compared to the control condition (white bar). However, there is a significant interaction of CsA and Anesthesia/Surgery on the number of entries, and treatment with CsA attenuates the Anesthesia/Surgery-induced decrease in the number of entries. CsA, cyclosporine A. ATP, adenosine triphosphate. N = 9 and 10 in each group of ATP studies and behavioral studies, respectively.

Given the findings that Anesthesia/Surgery was able to impair some behaviors in the mice, next, we assessed whether the Anesthesia/Surgery could induce energy deficits in the brain tissues (cortex). We found that the Anesthesia/Surgery (black bar) decreased brain (cortex) ATP levels as compared to the control condition (white bar): 67% versus 100%, P = 0.0002 (Fig. 5A) immediately after the Anesthesia/Surgery. Treatment with CsA alone did not significantly alter the brain ATP level as compared to the control condition; however, there was a significant interaction between CsA and the Anesthesia/Surgery on ATP level, and CsA attenuated the Anesthesia/Surgery-induced reduction in brain ATP level (F = 4.990, P = 0.033, Fig. 5A). Next, we found that the Anesthesia/Surgery (black bar) significantly increased brain ROS level as compared to the control condition (white bar): 133% versus 100%, P = 0.035 (Supplemental information Fig. 3) immediately after the Anesthesia/Surgery. Treatment with CsA alone did not significantly change the ROS levels, and CsA did not attenuate the Anesthesia/Surgery-induced ROS accumulation (F = 1.128, P = 0.301, Supplemental information Fig. 3). These findings suggest that the Anesthesia/Surgery was able to induce energy deficits and oxidative stress in brain tissues, and that CsA selectively attenuated the Anesthesia/Surgery-induced energy deficits without affecting oxidative stress.

Given the findings that CsA selectively attenuated the Anesthesia/Surgery-induced energy deficits, next, we determined whether CsA could also ameliorate the Anesthesia/Surgery-induced behavior changes. Two-way ANOVA showed that there was a significant interaction between CsA and the Anesthesia/Surgery on the latency to eat food in the buried food test, with CsA ameliorating the Anesthesia/Surgery-induced increase in the latency to eat food in the mice at 9 hours after the Anesthesia/Surgery (F = 6.497, P = 0.015, Fig. 5B). CsA also ameliorated the Anesthesia/Surgery-induced reduction in the number of entries in the novel arm in the Y maze test at 6 (F = 8.455, P = 0.006, Fig. 5C) and 9 (F = 27.24, P = 0.0001, Fig. 5D) hours after the Anesthesia/Surgery.

However, CsA ameliorated neither the Anesthesia/Surgery-induced reduction in the time spent in the center in the open field test (Supplemental information Fig. 4A, F = 0.573, P = 0.454) nor the Anesthesia/Surgery-induced reduction of the duration in the novel arm in the Y maze test at 6 hours (Supplemental information Fig. 4B, F = 0.732, P = 0.398) after the Anesthesia/Surgery in the mice. These data suggest that CsA could selectively ameliorate the Anesthesia/Surgery-induced behavior changes in the mice.

## Discussion

The goal of the current study was to develop a method of assessing behavior changes associated with delirium in the mouse using a combination of natural and learned behaviors, and then use this model to evaluate a hypothesized mechanism of delirium based on energy depletion. We found that the Anesthesia/Surgery impaired both natural and learned behavior of mice with acute onset and fluctuating course, and both of the Anesthesia/Surgery-induced behavioral changes and the Anesthesia/Surgery-induced reduction in ATP levels were ameliorated by CsA, an inhibitor of mitochondria permeability transient pore.

The CAM, a widely used tool in determining the presence of delirium in patients, includes: (1) acute onset and fluctuating course, (2) inattention, (3) disorganized thinking, and (4) altered level of consciousness. The assessment of delirium using CAM consists of many tests to determine the changes in both natural behavior (e.g., attention) and learned behavior (e.g., inability to remember events in the hospital or difficulty remembering instructions). A single behavior test in animals may not be sufficient enough to assess the aspects of delirium and is not consistent with the concept of CAM. Therefore, we set out to employ a battery of behavior tests to assess behavioral changes in mice.

We first found that the Anesthesia/Surgery increased the latency of mice to eat the food as compared to the control condition (white bar) in the buried food test (Fig. 2). Buried food test measures the motivation and ability of mice to eat food; it also tests the olfaction of the mice^14^,^15^. However, only mice with presence and intactness of attention, organized thinking, and consciousness were able to find and eat the food. Therefore, the Anesthesia/Surgery-induced impairment in mice’s ability to search for and eat food suggests that the Anesthesia/Surgery might cause the mice to develop the changes in behaviors (inattention, disorganized thinking and altered level of consciousness) associated with delirium.

Next, we found that the Anesthesia/Surgery did not significantly alter the total distance mice travelled in the open field test as compared to the control condition (Supplemental information Fig. 1). The total distance travelled indicates the locomotor activity of the mice^16^,^17^. Thus, these results suggest that the Anesthesia/Surgery in the current studies did not significantly impair the locomotor activity of the mice, and that the changes in behavior would not be due to the changes in the locomotor activity. This conclusion was further supported by the finding that the Anesthesia/Surgery did not significantly alter the number of arm visits in the Y maze test, as the number of arm visits also represents locomotor activity^18^,^19^ (Supplemental information Fig. 2).

Then, we found that the Anesthesia/Surgery decreased the time spent in the center (Fig. 3A1) and decreased the freezing time (Fig. 3B1) as compared to the control condition in the open field test at 6 hours after the Anesthesia/Surgery. These findings suggest that the Anesthesia/Surgery altered the natural behavior of the mice, e.g., anxiety (time spent in the center^20^,^21^,^22^) and natural reaction (freezing time^23^). Note that these behaviors also require the presence and intactness of attention, consciousness and organized thinking in the mice. Consistently, in humans, delirium may be associated with either hypervigilance or a reduced awareness of the environment.

Finally, the findings that the Anesthesia/Surgery decreased the number of entries (Fig. 4A1) and the duration of time in the novel arm (Fig. 4B1) suggest that the Anesthesia/Surgery impaired the spatial memory of the mice^24^,^25^,^26^, which also requires the presence and intactness of attention, consciousness and organized thinking. Consistently, delirious humans will often have impaired spatial memory, and walk into other people’s rooms, or forget the location of their bathroom, leading to continence problems.

Taken together, the Anesthesia/Surgery was able to impair certain natural and learned behaviors in mice that are dependent on attention, consciousness and organized thinking. The findings that the Anesthesia/Surgery only altered these behaviors at 6 or 9 hours, but not 24 hours, after the Anesthesia/Surgery suggest the acute onset of these changes. We purposely selected 6 and 9 hours, a close interval, to demonstrate the fluctuating course of such changes in behavior. We were able to show that some behavior changes only occurred at 6 hours (time spent in the center, freezing time, and duration in the novel arm); some occurred at 9, but not 6 or 24, hours (latency to eat food); and others occurred at both 6 and 9, but not 24, hours (entries in the novel arm) after the Anesthesia/Surgery (Table 1). These results showed the fluctuating course of these Anesthesia/Surgery-induced changes in behavior. Collectively, these findings suggest that the combination of these tests (buried food test, open field test and Y maze test) could be employed to serve as a battery of behavior tests in mice to establish an animal model capturing certain aspects of postoperative delirium consistent with the CAM algorithm. It is likely that the combination (battery) of other behavior tests may also be useful to demonstrate the aspects of postoperative delirium. Future studies to illustrate and optimize the battery of behavior tests for the animal model of postoperative delirium are warranted.

Moreover, we employed the clinical investigation method for calculating a composite Z score for each of 14 mice in both the control group and the Anesthesia/Surgery group (Table 2). We found that the composite Z scores of the mice in the control group varied more than those of the mice in the Anesthesia/Surgery group, and the mean of the composite Z score in the Anesthesia/Surgery mice was greater than that in the control group. Furthermore, the composite Z score of Anesthesia/Surgery mice at 9 (1.539), but not 6 (0.968), hours was greater than that at 24 hours (−0.054). Taken together, these findings suggest that the composite Z score could be used to determine the severity of changes in behavior in the mice, analogous to the CAM-S^27^ and other validated delirium severity measures in humans. Further investigations to validate this system (battery of behavior tests and the associated composite Z score) as an animal model to study postoperative delirium are warranted in the future.

The finding that the Anesthesia/Surgery did not significantly change the latency to the center (Fig. 3C) suggests that the Anesthesia/Surgery only impaired certain behaviors of the mice. These results may establish a system to study the selective neurotoxicity and neurobehavioral deficits caused by anesthesia and/or surgery in the future.

In the mechanistic studies, the Anesthesia/Surgery decreased cortex ATP levels (Fig. 5A) and increased ROS levels (Supplemental information Fig. 3) as compared to the control condition. These data suggest that the Anesthesia/Surgery was able to induce energy deficits and oxidative stress in the brain tissues of mice.

Opening of mitochondrial permeability transition pore (mPTP) can cause mitochondrial dysfunction, leading to reductions in mitochondrial membrane potential and decreases in the generation of adenosine-5′-triphosphate (ATP)^28^,^29^,^30^. Cyclosporine A (CsA) is an inhibitor of mPTP opening^31^,^32^,^33^,^34^,^35^,^36^,^37^,^38^ and has been shown to rescue the anesthetic isoflurane-induced opening of mPTP, caspase-3 activation and impairment of learning and memory in mice^39^. We therefore employed CsA in the current studies to determine whether the reduction in brain ATP level, associated with mitochondrial dysfunction, might contribute to the Anesthesia/Surgery-induced changes in mouse behavior. In the current studies, CsA selectively attenuated the Anesthesia/Surgery-induced reduction in ATP levels (Fig. 5A), but not the increase in ROS levels (Supplemental information Fig. 3). CsA also selectively ameliorated the Anesthesia/Surgery-induced increase in latency to eat food in the buried food test (Fig. 5B) and reduction in entries in the novel arm of Y maze test (Fig. 5C,D), but not the reduction in the amount of time spent in the center in the open field test (Supplemental information Fig. 4A) nor the reduction of duration in the novel arm (Supplemental information Fig. 4B) of Y maze test.

Taken together, these results suggest that energy deficits could be the underlying mechanism only for some of the Anesthesia/Surgery-induced impairment in behaviors (e.g., latency to eat food and entries in the novel arm). Consistently, the current studies demonstrated that Anesthesia/Surgery could reduce ATP level *in vivo* in brain tissues (cortex) of mice. Moreover, mPTP inhibitor CsA specifically mitigated the Anesthesia/Surgery-induced decrease in ATP levels and impairment of behaviors in buried food test and number of entries in Y maze test, but not the increases in ROS levels and impairment of behaviors in open field test and duration of time in Y maze test. Collectively, these findings suggest that the battery of behavioral test (buried food test, open field test and Y maze test) in mice could be used to study postoperative delirium in the mice. Furthermore, these results suggest that energy deficits (e.g., reduction in ATP levels) might contribute to some aspects of postoperative delirium pending further investigations.

Anesthesia and/or surgery has been shown to induce neuroinflammation^40^,^41^,^42^,^43^,^44^,^45^,^46^, Aβ accumulation^41^,^47^, and Tau phosphorylation^41^,^48^. Neuroinflammation^49^,^50^, Aβ accumulation^51^,^52^,^53^,^54^, and Tau phosphorylation^55^,^56^,^57^,^58^ have been reported to induce mitochondrial dysfunction, leading to energy deficit, e.g., reduction in ATP level. Moreover, CsA, the inhibitor of mPTP, was able to rescue the Anesthesia/Surgery-induced reduction in brain ATP level (Fig. 5). Collectively, these findings suggest that the Anesthesia/Surgery may reduce brain ATP level by causing mitochondria dysfunction. Further studies to test this hypothesis are warranted.

However, CsA is one of the immunosuppressants and thus has significant side effects^59^,^60^,^61^, which may impede its potential clinical application in treating and preventing the Anesthesia/Surgery-induced delirium. The findings that CsA attenuated the Anesthesia/Surgery-induced changes in behavior and reduction in ATP only suggest that energy deficit could contribute, at least partially, to the underlying mechanism of the Anesthesia/Surgery-induced delirium. Future studies would include the determination of whether other drugs, which can rescue energy deficit, e.g., Coenzyme Q10 or Vitamin K2^62^, can be used to prevent or treat the Anesthesia/Surgery-induced delirium.

The “paddling” T-maze alternation task^11^ and the attention set-shifting task (AST)^12^ have been reported as animal models of delirium. Note these studies use single or few learned behaviors in the mice. The current study, however, is the first one to combine several tests (battery of behavior tests) that utilize both natural and learned behaviors to establish an animal model to study postoperative delirium. Moreover, the establishment of a composite Z score based on these behavior changes would quantitatively determine the changes of behaviors associated with postoperative delirium in mice.

The current studies have several limitations. First, the buried food test might not be an absolute natural behavior, because we gave each mouse 2 pieces of the sweetened cereal two days before the first buried food test. However, the sweetened cereal served more to enable the mice to recognize food than to train the mice for the purpose of learning. Nevertheless, the mice would need attention, organized thinking and consciousness to perform the buried food test, as well as the open field test and the Y maze test. Second, we only determined the effects of Anesthesia/Surgery on the levels of ATP and ROS in the cortex of mice. The Anesthesia/Surgery could have different effects on the levels of ATP and ROS in different regions of the brain, e.g., hippocampus. Future investigations should look into the potential effects of Anesthesia/Surgery, and other perioperative factors, on the levels of ATP and ROS in other regions of the brain, utilizing our established system. Third, the pain following the Anesthesia/Surgery could a confounding factor in the data interpretation of the current studies because incision pain induces cognitive impairment in rodents^63^. However, the mice received EMLA cream (2.5% lidocaine and 2.5% prilocaine) for the treatment of pain in the current studies, and our previous studies have shown that EMLA was able to treat the pain induced by the surgical incision in mice^45^,^47^,^63^. Finally, we only employed 4-month-old female mice, but not the aged mice, in the studies. However, the main objective of the current studies was to establish a system to study postoperative delirium. Future research would include the comparison of the effects of the Anesthesia/Surgery on behavior changes in mice of different ages and sexes.

In conclusion, we found that the laparotomy under isoflurane anesthesia (Anesthesia/Surgery) in mice impaired behaviors in buried food test, open field test and Y maze test, which are dependent on the presence and intactness of attention, organized thinking and consciousness; these impairments were acute and fluctuating, and consistent with the CAM features of delirium. Moreover, we used a composite Z score to quantitatively describe these behaviors analogous to a delirium severity measurement. Given that the assessment of delirium in human includes (1) acute onset and fluctuating course, (2) inattention, (3) disorganized thinking, and (4) altered level of consciousness, the results in our current studies suggest that we might use the combination (battery of behavior tests) of buried food test, open field test and Y maze test to establish a system to study postoperative delirium in mice. We also found that the Anesthesia/Surgery selectively induced energy deficits and certain impairments in behavior that could be rescued by CsA, an mPTP inhibitor. Collectively, these findings demonstrate a potential animal model to study postoperative delirium in mice and suggest that energy deficits could contribute, at least partially, to postoperative delirium, pending further investigation.

## Methods

### Mouse surgery and treatment

All experiments were performed in accordance with the National Institutes of Health guidelines and regulations. The animal protocol was approved by the Massachusetts General Hospital (Boston, Massachusetts) Standing Committee on the Use of Animals in Research and Teaching. Efforts were made to minimize the number of animals used. C57BL/6J mice (4-month-old, female, The Charles River Laboratories, Wilmington, MA) were housed in a controlled environment (20–22 °C; 12 hours of light/dark on a reversed light cycle) for seven days prior to the studies. Given the fact that it is difficult and expensive to employ aged mice in the experiment, and the fact that delirium can occur in any age group, we used adult mice to conceptually establish the system. Similarly, at the current stage, we only employed same sex (e.g., female) of mice in the studies to establish a system, which could be used in the future to determine the effects of sex and age on the Anesthesia/Surgery-induced behavior changes in the established system. The mice were randomly assigned to the Anesthesia/Surgery group or the control group. The Anesthesia/Surgery was started between 6:00 and 7:00 am. A simple laparotomy was performed under isoflurane anesthesia using the methods described in our previous studies^64^. Specifically, anesthesia was induced and maintained with 1.4% isoflurane in 100% oxygen in a transparent acrylic chamber. Fifteen minutes after the induction, the mouse was moved out of the chamber, and isoflurane anesthesia was maintained via a cone device. One 16-gauge needle was inserted into the cone near the nose of the mouse to monitor the concentration of isoflurane. A longitudinal midline incision was made from the xiphoid to the 0.5 centimeter proximal pubic symphysis on the skin, abdominal muscles and peritoneum. Then, the incision was sutured layer by layer with 5–0 Vicryl thread. At the end of the procedure, EMLA cream (2.5% lidocaine and 2.5% prilocaine) was applied to the incision wound, and then every eight hours for two days to treat the pain associated with the incision. The procedure for each mouse lasted about ten minutes, and the mouse was put back into the anesthesia chamber for up to two hours to receive the rest of the anesthesia consisting of 1.4% isoflurane in 100% oxygen. The temperature of the anesthetizing chamber was controlled (DC Temperature Control System; FHC, Bowdoinham, Maine) to maintain the rectal temperature of the mice at 37 ± 0.5 °C during the Anesthesia/Surgery. After recovering from the anesthesia, each mouse was returned to a home cage with food and water available *ad libitum*. The mice in the control group were placed in their home cages with room air for two hours, which was consistent with the condition of non-surgery patients. Our previous studies found that neither this type of surgery^45^,^47^ nor anesthesia with 1.4% isoflurane^65^ significantly disturbed the blood pressure, blood gas values of the mice. EMLA was able to treat the pain associated with the surgery in the mice^45^,^47^. In the interventional studies, CsA [an inhibitor of mitochondrial permeability transition pore (mPTP) opening] (Sigma-Aldrich Inc., Natick, MA, Cat. Number: C3662) or saline was administered to mice via intraperitoneal injection 30 minutes before the control treatment or the Anesthesia/Surgery. The dose of CsA (10 mg/kg) was selected according to the previous studies^39^.

#### Behavior tests

As demonstrated in the diagram (Fig. 1), all mice had multiple behavioral tests in the order of buried food test, then open field test and finally Y maze test at 24 hours before (baseline) the Anesthesia/Surgery, and at 6, 9, and 24 hours after the Anesthesia/Surgery. We performed the behavior tests in groups of 3 mice and finished them within 50 minutes, which mimics the certain features of clinical evaluation of delirium in patients.

#### Buried food test

The buried food test was performed as described in previous studies^14^,^66^ with modifications. Specifically, two days before the test, we gave each mouse 2 pieces of the sweetened cereal. On all test days, we habituated the mice for one hour prior to the test by placing the home cage with mice in the testing room. The test cage was prepared with clean bedding (3 centimeters high). We buried 1 sweetened cereal pellet 0.5 centimeter below the surface of bedding so that it was not visible. The location of the food pellet was changed every time in a random fashion. We placed the mouse in the center of the test cage and measured the latency of the mouse to eat the food. Latency was defined as the time from when the mouse was placed in the test cage until when the mouse uncovered the food pellet and grasped it in the forepaws and/or teeth. Mice were allowed to consume the pellet they found and were then returned to their home cage. The observation time was 5 minutes. If the mouse could not find the pellet within 5 minutes, the testing session ended and the latency was defined as 300 seconds for that mouse. We emptied the bedding from the test cage and cleaned the cage with 70% ethanol solution after each test to prevent the transmission of olfactory cues. We changed gloves after each test.

#### Open field test

The open field test was performed as described in previous studies with modifications^67^,^68^. Specifically, the mouse was gently placed in the center of an open field chamber (40 × 40 × 40 centimeters) under dim light and was allowed to move freely for 5 minutes. The movement parameters of the mouse were monitored and analyzed via a video camera connected to the Any-Maze animal tracking system software (Stoelting Co., Wood Dale, IL). The total distance moved (meters), the time (seconds) spent in the center of the open field, the freezing time (seconds) and the latency (the time in seconds for the mice to reach to the location at the first attempt) to the center of the open field were recorded and analyzed. The floor of the open field was cleaned with 70% ethanol solution between each test.

#### Y maze test

The Y maze test was performed as described in the previous studies with modifications^18^,^69^. Specifically, the Y maze, made of gray polyvinylene, was placed in a quiet and illuminated room. Each maze consisted of three arms (8 × 30 × 15 centimeters, width × length × height), with an angle of 120 degrees between each arm. The three arms included the start arm, in which the mouse starts to explore (always open); the novel arm, which is blocked at the first trial, but opened at the second trial; and the other arm (always open). In the experiment, the start arm and other arm were designed randomly to avoid spatial memory error. The Y maze test consisted of 2 trials separated by an inter-trial interval (ITI). The first trial (training) was 10 minutes in duration, which allowed the mouse to explore 2 arms (the start arm and other arm) of the maze, with the novel arm being blocked. After a 2 hours (for the studies of 6 and 24 hours after the Anesthesia/Surgery) or 4 hours (for the studies of 9 hours after the Anesthesia/Surgery) ITI, the second trial (retention) was conducted. For the second trial, the mouse was placed back in the maze in the same start arm with free access to all 3 arms for 5 minutes. A video camera, which was linked to the Any-Maze animal tracking system software, was installed 60 centimeters above the chamber to monitor and analyze the number of entries and the time spent in each arm. The time spent in and entries into the novel arms indicated the spatial recognition memory (learned behavior). Each of the arms of the Y maze was cleaned with 70% ethanol solution between trials.

#### Brain tissue harvest, lysis and protein quantification

Different mice were used for the harvest of brain tissue and the studies of the determination of brain ATP and ROS levels. The brain tissues (cortex) of the mice were harvested immediately at the end of the Anesthesia/Surgery by decapitation. The harvested brain tissues were homogenized on ice using immunoprecipitation buffer (10 mM Tris-HCl, pH 7.4, 150 mM NaCl, 2 mM EDTA, 0.5% Nonidet P-40) plus protease inhibitors (1 mg/ml aprotinin, 1 mg/ml leupeptin, 1 mg/ml pepstatin A). The lysates were collected, centrifuged at 10,000 rpm for 5 minutes at 4 °C, and quantified for total proteins by bicinchoninic acid (BCA) protein assay kit (Pierce, Iselin, NJ). The brain tissues were then subjected to ATP and ROS measurement.

#### ATP Measurement

The levels of ATP in the cortex of mice (N = 9 in each group) were determined by the ATP Colorimetric/Fluorometric Assay Kit following the protocol provided by the manufacturer (Biovision Inc, Milpitas, CA) and the methods described in our previous studies^39^.

#### ROS Measurement

An OxiSelect *In Vitro* ROS/RNS Assay Kit (Cell Biolabs, San Diego, CA) was used to measure the amount of ROS in the cortex (N = 6 in each group), according to the protocols provided by the company and the methods described in our previous studies^39^.

#### Statistics

Data were expressed as mean ± standard error of mean (SEM). The number of samples was 10–14 per group for the behavior tests and 6–9 per group for the biochemical studies. The power calculation was performed using information collected from a preliminary study that was conducted under the same conditions. Based on the preliminary data, assuming a two-sided Student’s t-test, samples of 6 and 10 for each control and treatment group for the biochemistry and behavior studies, respectively, would lead to 90% power and 95% significance. In behavior tests, all the behavior parameters at 6, 9 and 24 hours were presented as a percentage of those of the baseline for the same group. We used the Mann-Whitney test to determine the difference in behavior tests between the control condition and Anesthesia/Surgery. In the intervention studies, normality of data was first analyzed by using the Shapiro–Wilk test, and we found the data were not normally distributed. Thus, logarithmic transformation was applied to normalize these variables. A two-way ANOVA was then used to assess the interaction of CsA with Anesthesia/Surgery to test the hypothesis that CsA would mitigate the effects of the Anesthesia/Surgery on behavior (e.g., latency to eat buried food in the buried food test) and levels of ATP and ROS in brain tissues of mice, followed by Tukey test for post-hoc comparisons. ATP and ROS levels were presented as a percentage of those of the control group. Z score was calculated using the formula described by Moller *et al*.^70^, Z = [***Δ**X*_*Anesthesia/Surgery*_− MEAN*(**Δ**X)*_control_]/SD*(**Δ**X)*_control_. In the formula, ***Δ**X*_control_ was the change score of mice in the control group at 6, 9 and 24 h after control condition minus the score at the baseline; ***Δ**X*_*Anesthesia/Surgery*_was the change score of mice in Anesthesia/Surgery group at 6, 9 and 24 h after the Anesthesia/Surgery minus the score at baseline; MEAN*(**Δ**X)*_control_ was the mean of ***Δ**X*_control_; and SD*(**Δ**X)*_control_ was the standard deviation of ***Δ**X*_control_. We also used the method for calculating a composite Z score in patients^71^,^72^ to determine a composite Z score for each of the mice. Specifically, the composite Z score for the mouse was calculated as the sum of the values of 6 Z scores (latency to eat food, time spent in the center, latency to the center, freezing time, entries in novel arm and duration in novel arm) normalized with the SD for that sum in the controls. Given that the reduction (rather than increase) in time spent in the center and the freezing time (open field test)^20^,^21^,^22^ and the reduction in duration and entries in the novel arm (Y maze test) indicate impairment of the behavior, we multiplied the Z score values representing these behaviors by −1 prior to calculating the composite Z score using these values. The nature of the hypothesis testing was two tailed. P values less than 0.05 were considered statistically significant. Prism 6 software (GraphPad Software, Inc, La Jolla, CA) was used to analyze the data.

## Additional Information

**How to cite this article**: Peng, M. *et al*. Battery of behavioral tests in mice to study postoperative delirium. *Sci. Rep.* **6**, 29874; doi: 10.1038/srep29874 (2016).

## Supplementary Material

Supplementary Information

## Figures and Tables

**Table 1:** Effects of the Anesthesia/Surgery on behavior in mice.

	**6Hr**	**9Hr**	**24Hr**
Buried food test			
Latency to eat food	—	↑	—
Open field test			
Total distance	—	—	—
Time spent in the center	↓	—	—
Freezing time	↓	—	—
Latency to the center	—	—	—
Y maze test			
Number of arm visits	—	—	—
Entries in novel arm	↓	↓	—
Duration in novel arm	↓	—	—

↑ and ↓ indicate the significant increase (*P* < 0.05) and decrease (*P* < 0.05) between the control condition and the Anesthesia /Surgery condition, respectively. The increases in the latency to eat food in the buried food test, and the latency to the center in the open field test suggest the impairment of behavior of the mice. The decreases in freezing time, the time spent in the center of the open field test, the entries in novel arm and the duration in novel arm in Y maze test suggest the impairment of behavior of the mice. Finally, the total distance in the open field test and number of arm visits in the Y maze test represent the function of locomotor activity of the mice.

**Table 2:** Summary of composite Z scores in Control and Anesthesia/Surgery mice.

**Mice**	**6Hr**	**9Hr**	**24Hr**
Control 1	0.328	−0.301	−0.154
Control 2	1.287	2.842	2.176
Control 3	0.586	0.045	0.444
Control 4	−0.966	−0.757	−0.883
Control 5	−1.758	0.219	0.318
Control 6	0.346	−0.519	−0.009
Control 7	−0.791	0.133	−0.918
Control 8	1.396	−0.739	−1.350
Control 9	0.764	−1.295	0.045
Control 10	−1.031	0.421	0.587
Control 11	−1.019	−0.401	1.500
Control 12	1.015	−0.313	0.146
Control 13	−0.553	1.056	−0.770
Control 14	0.395	−0.388	−1.134
**Control Mean**	**0.000**	**0.000**	**0.000**
**Control SEM**	**0.267**	**0.267**	**0.267**
Anesthesia/Surgery 1	0.715	0.389	−0.900
Anesthesia/Surgery 2	1.588	1.779	0.507
Anesthesia/Surgery 3	0.672	1.852	2.021
Anesthesia/Surgery 4	1.451	0.905	−3.114
Anesthesia/Surgery 5	0.748	1.188	−1.403
Anesthesia/Surgery 6	1.535	0.330	−0.128
Anesthesia/Surgery 7	2.696	0.747	0.843
Anesthesia/Surgery 8	−0.401	1.209	0.479
Anesthesia/Surgery 9	1.353	2.560	1.637
Anesthesia/Surgery 10	2.451	3.006	0.898
Anesthesia/Surgery 11	−0.815	0.356	0.143
Anesthesia/Surgery 12	0.119	0.576	−1.507
Anesthesia/Surgery 13	1.425	2.913	−0.078
Anesthesia/Surgery 14	0.014	3.741	−0.151
**Anesthesia/Surgery Mean**	**0.968**	**1.539**	**−0.054**
**Anesthesia/Surgery SEM**	**0.271**	**0.301**	**0.360**
P Value	0.017*	0.0007***	0.906

*P < 0.05; ***P < 0.001. The values of composite Z-score indicate the severity of the behavior impairment. The larger values of the composite Z score suggest severer impairment of the behavior of the mice.

## References

[b1] Marcantonio E. R. . A clinical prediction rule for delirium after elective noncardiac surgery. JAMA: the journal of the American Medical Association 271, 134–139 (1994). 8264068

[b2] Liu L. L. & Leung J. M. Predicting adverse postoperative outcomes in patients aged 80 years or older. Journal of the American Geriatrics Society 48, 405–412 (2000). 10798467 10.1111/j.1532-5415.2000.tb04698.x

[b3] Sieber F. E. & Barnett S. R. Preventing postoperative complications in the elderly. Anesthesiology clinics 29, 83–97 (2011). 21295754 10.1016/j.anclin.2010.11.011 PMC3073675

[b4] Whitlock E. L., Vannucci A. & Avidan M. S. Postoperative delirium. Minerva Anestesiol 77, 448–456 (2011). 21483389 PMC3615670

[b5] Inouye S. K. Delirium in older persons. The New England journal of medicine 354, 1157–1165 (2006). 16540616 10.1056/NEJMra052321

[b6] Saczynski J. S. . Cognitive trajectories after postoperative delirium. The New England journal of medicine 367, 30–39 (2012). 22762316 10.1056/NEJMoa1112923 PMC3433229

[b7] Ansaloni L. . Risk factors and incidence of postoperative delirium in elderly patients after elective and emergency surgery. The British journal of surgery 97, 273–280 (2010). 20069607 10.1002/bjs.6843

[b8] Jankowski C. J. . Cognitive and functional predictors and sequelae of postoperative delirium in elderly patients undergoing elective joint arthroplasty. Anesthesia and analgesia 112, 1186–1193 (2011). 21415433 10.1213/ANE.0b013e318211501b

[b9] Vasilevskis E. E., Han J. H., Hughes C. G. & Ely E. W. Epidemiology and risk factors for delirium across hospital settings. Best practice & research. Clinical anaesthesiology 26, 277–287 (2012). 23040281 10.1016/j.bpa.2012.07.003 PMC3580997

[b10] Deiner S. & Silverstein J. H. Postoperative delirium and cognitive dysfunction. British journal of anaesthesia 103 Suppl 1, i41–46 (2009). 20007989 10.1093/bja/aep291 PMC2791855

[b11] Murray C. . Systemic inflammation induces acute working memory deficits in the primed brain: relevance for delirium. Neurobiology of aging 33, 603–616 (2012). 20471138 10.1016/j.neurobiolaging.2010.04.002 PMC3200140

[b12] Culley D. J. . Systemic inflammation impairs attention and cognitive flexibility but not associative learning in aged rats: possible implications for delirium. Frontiers in aging neuroscience 6, 107 (2014). 24959140 10.3389/fnagi.2014.00107 PMC4050637

[b13] Inouye S. K. . Clarifying confusion: the confusion assessment method. A new method for detection of delirium. Annals of internal medicine 113, 941–948 (1990). 2240918 10.7326/0003-4819-113-12-941

[b14] Lehmkuhl A. M., Dirr E. R. & Fleming S. M. Olfactory assays for mouse models of neurodegenerative disease. J Vis Exp. e51804 (2014). 25177842 10.3791/51804 PMC4827975

[b15] Yang M. & Crawley J. N. Simple behavioral assessment of mouse olfaction. Curr Protoc Neurosci Chapter 8, Unit 8 24 (2009). 10.1002/0471142301.ns0824s48 PMC2753229 19575474

[b16] van Zyl P. J., Dimatelis J. J. & Russell V. A. Behavioural and biochemical changes in maternally separated Sprague-Dawley rats exposed to restraint stress. Metab Brain Dis 31, 121–133 (2016). 26555398 10.1007/s11011-015-9757-y

[b17] Dulawa S. C., Holick K. A., Gundersen B. & Hen R. Effects of chronic fluoxetine in animal models of anxiety and depression. Neuropsychopharmacology 29, 1321–1330 (2004). 15085085 10.1038/sj.npp.1300433

[b18] Rayatnia F. . Nitric oxide involvement in consolidation, but not retrieval phase of cognitive performance enhanced by atorvastatin in mice. European journal of pharmacology 666, 122–130 (2011). 21620819 10.1016/j.ejphar.2011.05.017

[b19] Dellu F., Mayo W., Cherkaoui J., Le Moal M. & Simon H. A two-trial memory task with automated recording: study in young and aged rats. Brain research 588, 132–139 (1992). 1393562 10.1016/0006-8993(92)91352-f

[b20] Ketcha Wanda G. J., Djiogue S., Gamo F. Z., Ngitedem S. G. & Njamen D. Anxiolytic and sedative activities of aqueous leaf extract of Dichrocephala integrifolia (Asteraceae) in mice. J Ethnopharmacol 176, 494–498 (2015). 26602454 10.1016/j.jep.2015.11.035

[b21] Bahi A., Schwed J. S., Walter M., Stark H. & Sadek B. Anxiolytic and antidepressant-like activities of the novel and potent non-imidazole histamine H(3) receptor antagonist ST-1283. Drug Des Devel Ther 8, 627–637 (2014). 10.2147/DDDT.S63088 PMC4044994 24920886

[b22] Prut L. & Belzung C. The open field as a paradigm to measure the effects of drugs on anxiety-like behaviors: a review. European journal of pharmacology 463, 3–33 (2003). 12600700 10.1016/s0014-2999(03)01272-x

[b23] Bagewadi H. G., Ak A. K. & Shivaramegowda R. M. An Experimental Study to Evaluate the Effect of Memantine in Animal Models of Anxiety in Swiss Albino Mice. J Clin Diagn Res 9, FF01–05 (2015). 26435964 10.7860/JCDR/2015/13233.6287 PMC4576556

[b24] Sun H., Mao Y., Wang J. & Ma Y. Effects of beta-adrenergic antagonist, propranolol on spatial memory and exploratory behavior in mice. Neuroscience letters 498, 133–137 (2011). 21571035 10.1016/j.neulet.2011.04.076

[b25] Akwa Y., Ladurelle N., Covey D. F. & Baulieu E. E. The synthetic enantiomer of pregnenolone sulfate is very active on memory in rats and mice, even more so than its physiological neurosteroid counterpart: distinct mechanisms? Proceedings of the National Academy of Sciences of the United States of America 98, 14033–14037 (2001). 11717462 10.1073/pnas.241503698 PMC61162

[b26] Javadi-Paydar M. . Atorvastatin improved scopolamine-induced impairment in memory acquisition in mice: involvement of nitric oxide. Brain research 1386, 89–99 (2011). 21354117 10.1016/j.brainres.2011.02.057

[b27] Inouye S. K. . The CAM-S: development and validation of a new scoring system for delirium severity in 2 cohorts. Annals of internal medicine 160, 526–533 (2014). 24733193 10.7326/M13-1927 PMC4038434

[b28] Fulda S., Galluzzi L. & Kroemer G. Targeting mitochondria for cancer therapy. Nature reviews. Drug discovery 9, 447–464 (2010). 20467424 10.1038/nrd3137

[b29] Kroemer G., Galluzzi L. & Brenner C. Mitochondrial membrane permeabilization in cell death. Physiol Rev 87, 99–163 (2007). 17237344 10.1152/physrev.00013.2006

[b30] Sullivan P. G., Rabchevsky A. G., Waldmeier P. C. & Springer J. E. Mitochondrial permeability transition in CNS trauma: cause or effect of neuronal cell death? J Neurosci Res 79, 231–239 (2005). 15573402 10.1002/jnr.20292

[b31] Fournier N., Ducet G. & Crevat A. Action of cyclosporine on mitochondrial calcium fluxes. J Bioenerg Biomembr 19, 297–303 (1987). 3114244 10.1007/BF00762419

[b32] Bernardi P. The permeability transition pore. Control points of a cyclosporin A-sensitive mitochondrial channel involved in cell death. Biochimica et biophysica acta 1275, 5–9 (1996). 8688451 10.1016/0005-2728(96)00041-2

[b33] Hansson M. J. . Brain-derived respiring mitochondria exhibit homogeneous, complete and cyclosporin-sensitive permeability transition. Journal of neurochemistry 89, 715–729 (2004). 15086528 10.1111/j.1471-4159.2004.02400.x

[b34] He L. & Lemasters J. J. Regulated and unregulated mitochondrial permeability transition pores: a new paradigm of pore structure and function? FEBS letters 512, 1–7 (2002). 11852041 10.1016/s0014-5793(01)03314-2

[b35] Nicolli A., Basso E., Petronilli V., Wenger R. M. & Bernardi P. Interactions of cyclophilin with the mitochondrial inner membrane and regulation of the permeability transition pore, and cyclosporin A-sensitive channel. The Journal of biological chemistry 271, 2185–2192 (1996). 8567677 10.1074/jbc.271.4.2185

[b36] Norman K. G. . Cyclosporine A suppresses keratinocyte cell death through MPTP inhibition in a model for skin cancer in organ transplant recipients. Mitochondrion 10, 94–101 (2010). 19836469 10.1016/j.mito.2009.10.001 PMC6917037

[b37] Alessandri B. . Cyclosporin A improves brain tissue oxygen consumption and learning/memory performance after lateral fluid percussion injury in rats. Journal of neurotrauma 19, 829–841 (2002). 12184853 10.1089/08977150260190429

[b38] Osman M. M. . Cyclosporine-A as a neuroprotective agent against stroke: Its translation from laboratory research to clinical application. Neuropeptides 45, 359–368 (2011). 21592568 10.1016/j.npep.2011.04.002

[b39] Zhang Y. . Anesthetics isoflurane and desflurane differently affect mitochondrial function, learning, and memory. Annals of neurology 71, 687–698 (2012). 22368036 10.1002/ana.23536 PMC3942786

[b40] Wan Y. . Postoperative impairment of cognitive function in rats: a possible role for cytokine-mediated inflammation in the hippocampus. Anesthesiology 106, 436–443 (2007). 17325501 10.1097/00000542-200703000-00007

[b41] Wan Y. . Cognitive decline following major surgery is associated with gliosis, beta-amyloid accumulation, and tau phosphorylation in old mice. Critical care medicine 38, 2190–2198 (2010). 20711073 10.1097/CCM.0b013e3181f17bcb

[b42] Cibelli M. . Role of interleukin-1beta in postoperative cognitive dysfunction. Annals of neurology 68, 360–368 (2010). 20818791 10.1002/ana.22082 PMC4836445

[b43] Terrando N. . Tumor necrosis factor-alpha triggers a cytokine cascade yielding postoperative cognitive decline. Proceedings of the National Academy of Sciences of the United States of America 107, 20518–20522 (2010). 21041647 10.1073/pnas.1014557107 PMC2996666

[b44] Wu X. . The inhalation anesthetic isoflurane increases levels of proinflammatory TNF-alpha, IL-6, and IL-1beta. Neurobiology of aging 33, 1364–1378 (2012). 21190757 10.1016/j.neurobiolaging.2010.11.002 PMC3117127

[b45] Xu Z. . Peripheral surgical wounding and age-dependent neuroinflammation in mice. PloS one 9, e96752 (2014). 24796537 10.1371/journal.pone.0096752 PMC4010504

[b46] Zhang L. . Isoflurane and sevoflurane increase interleukin-6 levels through the nuclear factor-kappa B pathway in neuroglioma cells. British journal of anaesthesia 110 Suppl 1, i82–91 (2013). 23604542 10.1093/bja/aet115 PMC3667345

[b47] Xu Z. . Age-dependent postoperative cognitive impairment and Alzheimer-related neuropathology in mice. Scientific reports 4, 3766 (2014). 24441878 10.1038/srep03766 PMC3895908

[b48] Dong Y., Wu X., Xu Z., Zhang Y. & Xie Z. Anesthetic isoflurane increases phosphorylated tau levels mediated by caspase activation and Abeta generation. PloS one 7, e39386 (2012). 22745746 10.1371/journal.pone.0039386 PMC3379981

[b49] Hunter R. L. . Inflammation induces mitochondrial dysfunction and dopaminergic neurodegeneration in the nigrostriatal system. Journal of neurochemistry 100, 1375–1386 (2007). 17254027 10.1111/j.1471-4159.2006.04327.x

[b50] Witte M. E., Geurts J. J., de Vries H. E., van der Valk P. & van Horssen J. Mitochondrial dysfunction: a potential link between neuroinflammation and neurodegeneration? Mitochondrion 10, 411–418 (2010). 20573557 10.1016/j.mito.2010.05.014

[b51] Eckert A. . Elevated vulnerability to oxidative stress-induced cell death and activation of caspase-3 by the Swedish amyloid precursor protein mutation. J Neurosci Res 64, 183–192 (2001). 11288146 10.1002/jnr.1064

[b52] Leutz S. . Reduction of trophic support enhances apoptosis in PC12 cells expressing Alzheimer’s APP mutation and sensitizes cells to staurosporine-induced cell death. J Mol Neurosci 18, 189–201 (2002). 12059037 10.1385/JMN:18:3:189

[b53] Marques C. A. . Neurotoxic mechanisms caused by the Alzheimer’s disease-linked Swedish amyloid precursor protein mutation: oxidative stress, caspases, and the JNK pathway. The Journal of biological chemistry 278, 28294–28302 (2003). 12730216 10.1074/jbc.M212265200

[b54] Keil U. . Amyloid beta-induced changes in nitric oxide production and mitochondrial activity lead to apoptosis. The Journal of biological chemistry 279, 50310–50320 (2004). 15371443 10.1074/jbc.M405600200

[b55] Ebneth A., Drewes G., Mandelkow E. M. & Mandelkow E. Phosphorylation of MAP2c and MAP4 by MARK kinases leads to the destabilization of microtubules in cells. Cell Motil Cytoskeleton 44, 209–224 (1999). 10542369 10.1002/(SICI)1097-0169(199911)44:3<209::AID-CM6>3.0.CO;2-4

[b56] Gotz J., Ittner L. M., Fandrich M. & Schonrock N. Is tau aggregation toxic or protective: a sensible question in the absence of sensitive methods? Journal of Alzheimer’s disease: JAD 14, 423–429 (2008). 18688093 10.3233/jad-2008-14410

[b57] Ittner L. M. . Dendritic function of tau mediates amyloid-beta toxicity in Alzheimer’s disease mouse models. Cell 142, 387–397 (2010). 20655099 10.1016/j.cell.2010.06.036

[b58] Stamer K., Vogel R., Thies E., Mandelkow E. & Mandelkow E. M. Tau blocks traffic of organelles, neurofilaments, and APP vesicles in neurons and enhances oxidative stress. The Journal of cell biology 156, 1051–1063 (2002). 11901170 10.1083/jcb.200108057 PMC2173473

[b59] Canafax D. M. & Ascher N. L. Cyclosporine immunosuppression. Clin Pharm 2, 515–524 (1983). 6360494

[b60] Sutton S., Cohen A. M. & Resnick M. I. Value of chest computed tomography in genitourinary malignancies. Urology 22, 667–668 (1983). 6685937 10.1016/0090-4295(83)90325-4

[b61] Ptachcinski R. J., Burckart G. J. & Venkataramanan R. Cyclosporine. Drug Intell Clin Pharm 19, 90–100 (1985). 3882378 10.1177/106002808501900202

[b62] Vos M. . Vitamin K2 is a mitochondrial electron carrier that rescues pink1 deficiency. Science 336, 1306–1310 (2012). 22582012 10.1126/science.1218632

[b63] Zhang X. . Surgical Incision-Induced Nociception Causes Cognitive Impairment and Reduction in Synaptic NMDA Receptor 2B in Mice. The Journal of neuroscience: the official journal of the Society for Neuroscience 33, 17737–17748 (2013). 24198365 10.1523/JNEUROSCI.2049-13.2013 PMC3818549

[b64] Ren Q. . Surgery plus anesthesia induces loss of attention in mice. Frontiers in cellular neuroscience 9, 346 (2015). 26441522 10.3389/fncel.2015.00346 PMC4561675

[b65] Xie Z. . The common inhalation anesthetic isoflurane induces caspase activation and increases amyloid beta-protein level *in vivo*. Annals of neurology 64, 618–627 (2008). 19006075 10.1002/ana.21548 PMC2612087

[b66] Nathan B. P., Yost J., Litherland M. T., Struble R. G. & Switzer P. V. Olfactory function in apoE knockout mice. Behavioural brain research 150, 1–7 (2004). 15033273 10.1016/S0166-4328(03)00219-5

[b67] Anchan D., Clark S., Pollard K. & Vasudevan N. GPR30 activation decreases anxiety in the open field test but not in the elevated plus maze test in female mice. Brain Behav 4, 51–59 (2014). 24653954 10.1002/brb3.197 PMC3937706

[b68] Li X. M. . Disruption of hippocampal neuregulin 1-ErbB4 signaling contributes to the hippocampus-dependent cognitive impairment induced by isoflurane in aged mice. Anesthesiology 121, 79–88 (2014). 24589481 10.1097/ALN.0000000000000191 PMC4062586

[b69] Chen Y. . Anxiety- and depressive-like behaviors in olfactory deficient Cnga2 knockout mice. Behavioural brain research 275, 219–224 (2014). 25192635 10.1016/j.bbr.2014.08.042

[b70] Moller J. T. . Long-term postoperative cognitive dysfunction in the elderly ISPOCD1 study. ISPOCD investigators. International Study of Post-Operative Cognitive Dysfunction. Lancet 351, 857–861 (1998). 9525362 10.1016/s0140-6736(97)07382-0

[b71] Voigt Hansen M., Rasmussen L. S., Jespersgaard C., Rosenberg J. & Gogenur I. There is no association between the circadian clock gene HPER3 and cognitive dysfunction after noncardiac surgery. Anesthesia and analgesia 115, 379–385 (2012). 22543063 10.1213/ANE.0b013e318253d6b3

[b72] Rasmussen L. S. . The assessment of postoperative cognitive function. Acta Anaesthesiol Scand 45, 275–289 (2001). 11207462 10.1034/j.1399-6576.2001.045003275.x

