# Supplementary material for: Battery of behavioral tests in mice to study postoperative delirium
Source: Sci Rep. 2016 Jul 20;6:29874. doi: 10.1038/srep29874 (PMC4951688; doi:10.1038/srep29874)
Supplement: Supplementary Information [file srep29874-s1.pdf]

Supplemental information.

Battery of behavioral tests in mice to study postoperative delirium

Mian Peng, Ce Zhang, Yuanlin Dong, Yiying Zhang,
Harumasa Nakazawa, Masao Kaneki, Hui Zheng, Yuan Shen,
Edward R. Marcantonio and Zhongcong Xie

Supplemental information Figure 1.

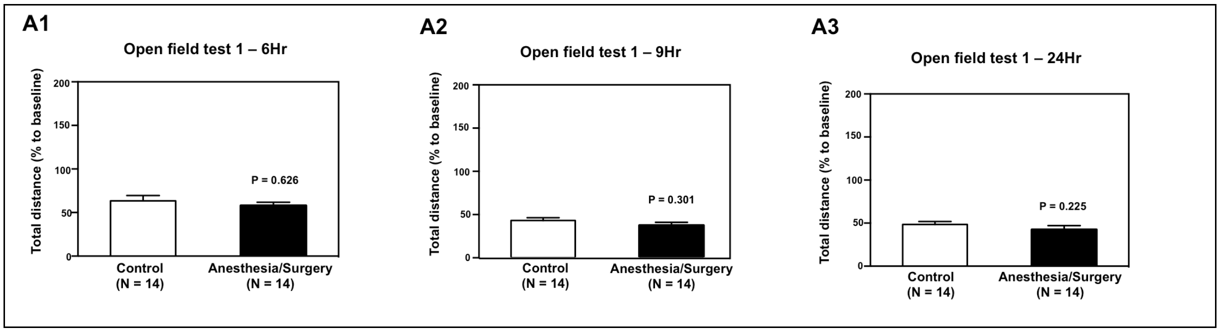

Supplemental information Figure 1. Effects of Anesthesia/Surgery on the behavior of mice in the open field test.

A. Anesthesia/Surgery (black bar) does not significantly change the total distance of movement of the mice in the open field test as compared to the control condition (white bar) at 6 (**A1**), 9 (**A2**) and 24 (**A3**) hours after the Anesthesia/Surgery.

Supplemental information Figure 2.

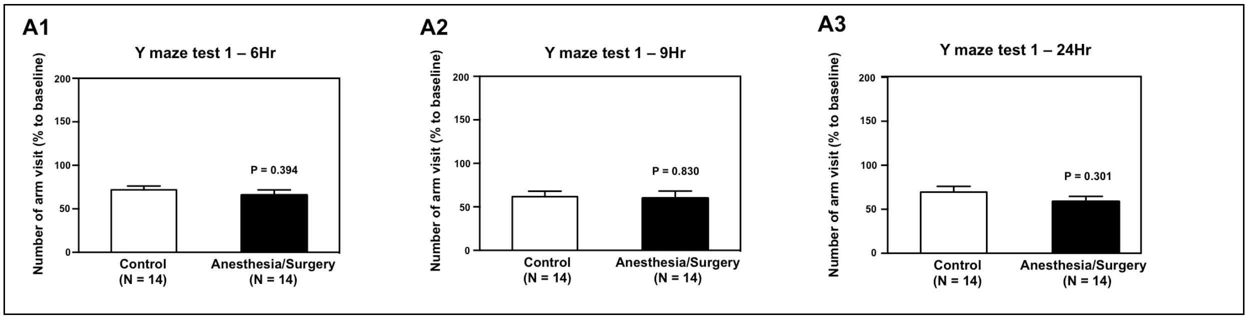

Supplemental information Figure 2. Effects of Anesthesia/Surgery on the behavior of mice in Y maze test. A. Anesthesia/Surgery (black bar) does not significantly change the number of arm visits in the Y maze test as compared to the control condition (white bar) at 6 (**A1**), 9 (**A2**) and 24 (**A3**) hours after the Anesthesia/Surgery in the mice.

Supplemental information Figure 3.

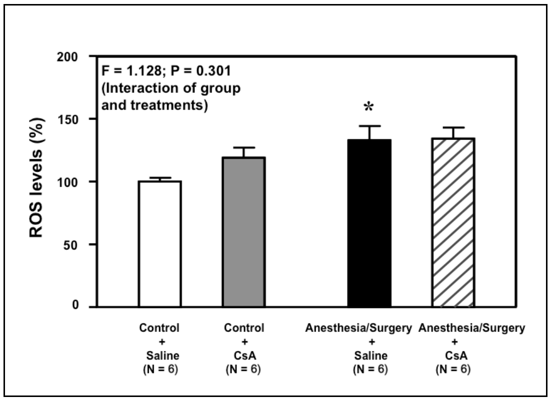

Supplemental information Figure 3. CsA does not attenuate the Anesthesia/Surgery-induced ROS accumulation. Anesthesia/Surgery (black bar) increases the ROS levels in mouse brain tissues as compared to the control condition (white bar) immediately after the Anesthesia/Surgery. Treatment with CsA alone (gray bar) does not significantly change the ROS levels as compared to the control condition (white bar). There is no significant interaction of CsA and Anesthesia/Surgery on the ROS levels, and treatment with CsA does not attenuate the Anesthesia/Surgery-induced ROS accumulation. CsA, cyclosporine A; ROS, reactive oxygen species. N = 6 in each group.

Supplemental information Figure 4.

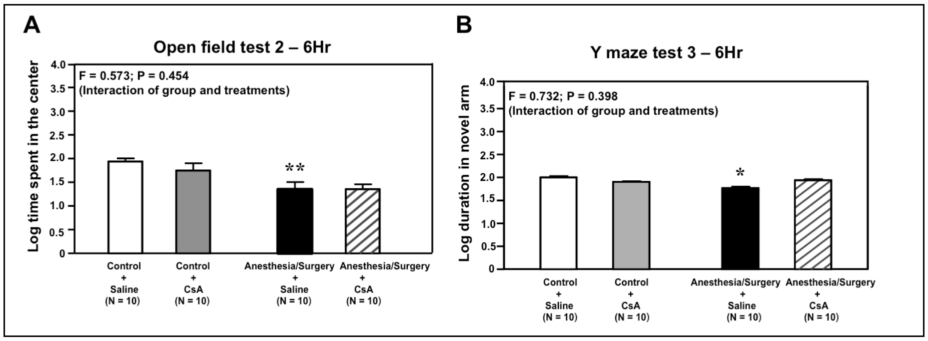

Supplemental information Figure 4. CsA does not mitigate the Anesthesia/Surgery-induced behavior changes in the open field test (time spent in the center) and Y maze test (duration). A. Anesthesia/Surgery (black bar) decreases the time spent in the center of open field test as compared to the control condition (white bar) at 6 hours after the Anesthesia/Surgery. Treatment with CsA alone (gray bar) does not significantly change the time spent in the center as compared to the control condition (white bar). There is no significant interaction of CsA and Anesthesia/Surgery on the time spent in the center, and treatment with CsA does not attenuate the Anesthesia/Surgery-induced decrease in the time spent in the center. **B.** Anesthesia/Surgery (black bar) decreases the duration in the novel arm of Y maze test as compared to the control condition (white bar) at 6 hours after the Anesthesia/Surgery. Treatment with CsA alone (gray bar) does not significantly change the duration as compared to the control condition (white bar). There is no significant interaction of CsA and Anesthesia/Surgery on the duration, and treatment with CsA does not attenuate the Anesthesia/Surgery-induced decrease in the duration. CsA, cyclosporine A. N = 10 in each group.
